# Caregiver-Reported Sugar-Sweetened Beverage Consumption and Cavities in Children Aged 1 to 5 Years, National Survey of Children’s Health 2021–2022

**DOI:** 10.5888/pcd22.250183

**Published:** 2025-09-11

**Authors:** Alexander H.W. Molinari, Mary Ellen Grap, Samantha L. Pierce, Ann Goding Sauer, Brook Belay, Alyson B. Goodman, Carrie Dooyema

**Affiliations:** 1 Division of Nutrition, Physical Activity, and Obesity, Centers for Disease Control and Prevention, Atlanta, Georgia; 2 US Public Health Service Commissioned Corps, Rockville, Maryland; 3 Epidemic Intelligence Service, Centers for Disease Control and Prevention, Atlanta, Georgia; 4 Oak Ridge Institute for Science and Education, Oak Ridge, Tennessee

## Abstract

**Introduction:**

Dental cavities are a common chronic disease among US children. Sugar-sweetened beverages (SSBs) are the leading contributor of added sugars in children’s diets. We assessed the prevalence and adjusted odds of a caregiver-reported cavity in the past 12 months by SSB consumption among children aged 1 to 5 years.

**Methods:**

We used data from the 2021–2022 National Survey of Children’s Health. Our sample comprised children aged 1 to 5 years who had seen an oral health provider in the past 12 months. An adult caregiver reported whether the child had a cavity in the past 12 months and how frequently they consumed SSBs in the past 7 days. Models were adjusted for age, sex, race and ethnicity, highest level of education among adults in the household, and household federal poverty level. We used multivariable logistic regression to examine the relationship between cavities and SSB consumption.

**Results:**

Among 23,023 US children in our sample, 11.6% had a caregiver-reported cavity in the past 12 months. Approximately 37.3% of children were reported to drink no SSBs, 39.5% drank SSBs 1 to 3 times in the past week, and 23.3% drank SSBs 4 or more times in the past week. Compared with children who drank no SSBs, those who drank SSBs 1 to 3 times in the past week or 4 or more times in the past week had 1.7 (95% CI, 1.4–2.2) times and 2.8 (95% CI, 2.1–3.6) times higher adjusted odds, respectively, to have a caregiver-reported cavity.

**Conclusion:**

Frequent SSB consumption is common among children aged 1 to 5 years and is associated with higher odds of having a cavity. These findings highlight the need for public health and dental professionals to promote the reduction of SSB intake and encourage healthier choices among very young children and their caregivers to help mitigate oral health issues.

**Summary**

**What is already known on this topic?**

Sugar-sweetened beverages (SSBs) are linked to dental cavities in US children, but data on very young children are limited.

**What is added by this report?**

This study used a large, nationally representative dataset to examine associations between SSB consumption and caregiver-reported cavities in children aged 1 to 5 years.

**What are the implications for public health practice?**

Findings underscore the need for public health initiatives to reduce SSB intake among very young children to improve oral health outcomes and prevent cavities.

## Introduction

Cavities, also known as tooth decay and dental caries, are a common chronic disease among US children and adolescents (1). Cavities refer to tooth decay that results from a dissolving process involving the hard outer layer of the tooth, the enamel (1). This process is caused by bacteria that metabolize sugar ingested from foods and drinks and produce acid that damages teeth (1). The National Health and Nutrition Examination Survey (NHANES) 2011–2016 showed that 23% of children aged 2 to 5 years and 52% of children aged 6 to 8 years had at least 1 cavity in a primary tooth (2). By ages 12 to 19 years, 57% of adolescents have at least 1 cavity in a permanent tooth (2).

Cavities, left untreated, can lead to infection, tooth loss, and affect a child’s ability to eat, sleep, and learn (3). To prevent cavities and other oral health problems, the American Academy of Pediatric Dentists recommends having routine dental visits starting by 1 year of age (4). The treatment of cavities also has economic ramifications. A study of US children receiving operating room or ambulatory surgical care for potentially preventable oral health conditions reported total Medicaid payments of $68 million in 2011, with an average cost of $2,581 per case (5). Most of these cases were for the treatment of cavities in children aged 1 to 5 years (5).

The formation of cavities can be influenced by various factors, including genetics, poor oral hygiene, lack of preventive dental care, dietary behaviors including sugary drink and food intake, and sociodemographic characteristics, such as household income level (6). NHANES data from 2015–2018 showed that sugar-sweetened beverages (SSBs) were the leading contributor of daily added sugars among children aged 2 to 19 years (7). The *2015–2020 Dietary Guidelines for Americans* defines SSBs as various liquids that contain added sugars, such as sucrose, dextrose, and high-fructose corn syrup (8). The 2021 National Survey of Children’s Health (NSCH) showed that SSB consumption is common even in very young children (aged 1–5 years), with nearly 60% of US children aged 1 to 5 years consuming at least 1 SSB in the past week (9). Similarly, NHANES data from 2011–2014 reported that just over 60% of young children (aged 2–5 years) consumed any amount of SSBs on a given day (10). This level of consumption is misaligned with the *2020–2025 Dietary Guidelines for Americans*, which state that children under 2 years of age should consume no added sugars and children 2 years or older should consume no more than 10% of their daily caloric intake in added sugars (11).

Multiple studies have shown a positive association between frequent consumption of SSBs and the presence of cavities in children and adults (10,12–15). For example, a study using 2011–2014 NHANES data found that SSB consumption was associated with cavities in young children (aged 2–5 years) and an increased risk of developing untreated cavities in all age groups (aged 2–74 years) (10). Furthermore, another study showed that SSB consumption can affect future oral health: frequent consumption of SSBs at age 10 to 12 months significantly increased the likelihood of having cavities at age 6 years (16). These studies highlight the important link between SSB consumption and dental cavities.

The most recent study, published in 2020, that used data from a national survey to examine the relationship between cavities and SSBs in young US children (aged 2–5 years), used data from over a decade ago (NHANES 2011–2014) (10). We examined the prevalence and adjusted odds of having a cavity by frequency of SSB consumption among US children aged 1 to 5 years by using nationally representative data from the 2021–2022 NSCH. The NSCH dataset is unique in that it includes children as young as 1 year of age, and it comprises a very large sample of children. Our study had a sample size of more than 23,000 children aged 1 to 5 years, whereas the NHANES 2011–2014 study had a sample size of 1,490 children aged 2 to 5 years. To our knowledge, our study is the first to use the NSCH dataset to quantify the relationship between cavities and SSBs in very young US children.

## Methods

NSCH is a cross-sectional nationally representative survey sponsored and directed by the Maternal and Child Health Bureau of the Health Resources and Services Administration (17,18). The NSCH has been conducted annually since 2016 and collects data on the physical and mental health, as well as the health care needs, of noninstitutionalized US children aged 0 to 17 years (17,18).

Home addresses are randomly selected for sampling from a list of all mailable addresses in the nation via the US Census Bureau’s Census Master Address File (17,18). Self-administered web-based and paper-based questionnaires are sent to the selected addresses for data collection (17,18). An adult caregiver living in the same household as the sample child is responsible for completing the voluntary survey, which is available in both English and Spanish (17,18). Because deidentified data from public sources were used, this research was considered exempt from institutional review board approval, and informed consent from subjects was not required.

**Figure 1:**
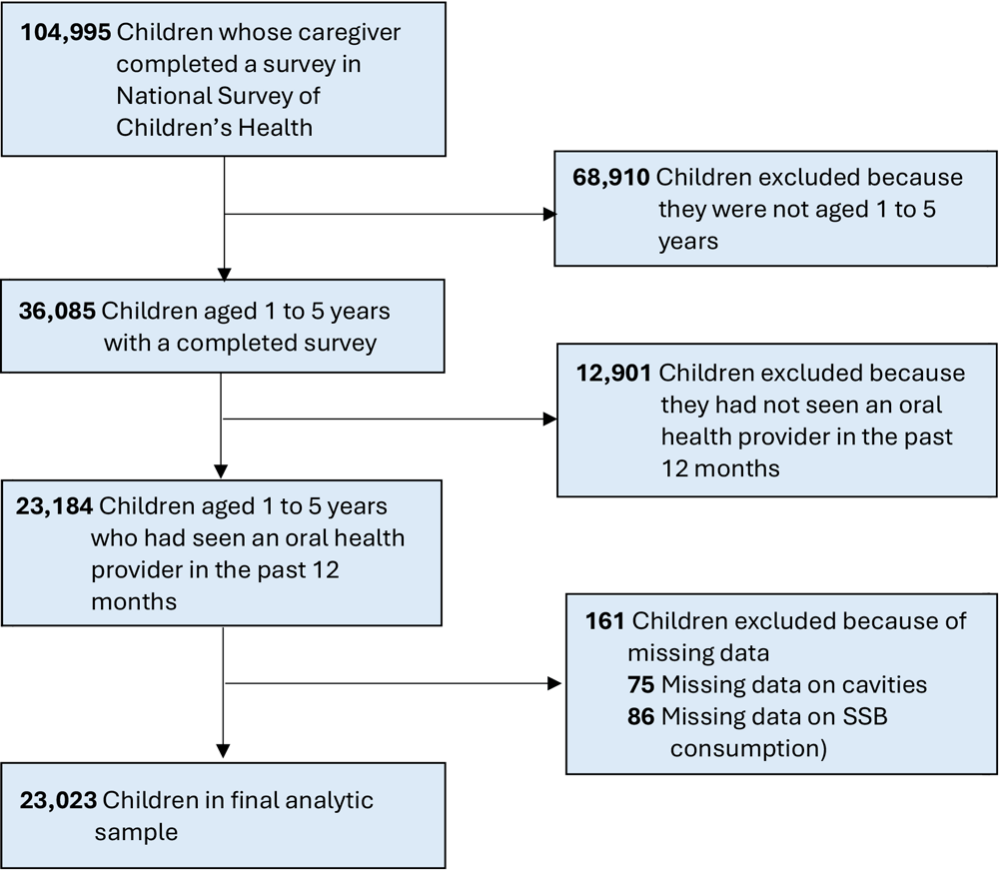
Inclusion criteria for final analytical sample in a study on the relationship between consumption of SSBs and cavities among US children aged 1 to 5 years. Data source: National Survey of Children’s Health 2021–2022. Abbreviation: SSB, sugar-sweetened beverage.

A total of 104,995 children aged 0 to 17 years had a caregiver who completed the 2021 and 2022 NSCH surveys. The overall weighted response rate was 40.3% in 2021 and 39.1% in 2022 (19). The combined dataset was weighted to represent the population of noninstitutionalized US children aged 0 to 17 years who live in housing units, nationally and in each state (19). Of the 104,995 children with complete surveys, 36,085 were children aged 1 to 5 years. Of these, 23,184 (64%) had seen an oral health provider in the past 12 months and were included in the study. The decision to include only children who had seen an oral health provider ensured that we included only children who had the opportunity to have any cavities diagnosed. Of note, the NSCH does not specify a definition for oral health provider, allowing for broad interpretation by the caregiver. Our final analytical sample included 23,023 children with complete data (Figure 1).

The outcome variable for this study was the presence of a caregiver-reported cavity for the child in the past 12 months. The following survey item was used to determine the outcome: “During the past 12 months, has this child had decayed teeth or cavities?” Caregivers were able to select yes or no. The main exposure variable of interest was SSB consumption, for which caregivers were asked, “During the past week, how many times did this child drink sugary drinks?” Instructions were provided by NSCH to not include 100% fruit juice, aligning with similar studies that focused only on beverages with added sugars (10).

Caregivers could choose from 6 mutually exclusive survey response options for the question on frequency of SSB consumption, which were collapsed into 3 groups: none, 1 to 3 times, or 4 or more times in the past week. Additional covariates were age (1–3 years [12 to <48 months], 4 years [48 to <60 months], or 5 years [60 to <72 months]), sex (male or female), race and ethnicity (Hispanic, non-Hispanic Black, non-Hispanic White, or non-Hispanic “Other” race [included Asian, American Indian/Alaska Native, Native Hawaiian and other Pacific Islander, and multiple races]), caregivers’ highest education level (high school graduate/General Education Development certificate or less, some college or technical school, or college degree or higher), and household federal poverty level (FPL) (<130%, 130% to <350%, or ≥350% FPL). We grouped together children aged 1 to 3 years to enhance statistical power, because the prevalence of reported cavities was low in each age within this range. Additionally, the NSCH uses sequential regression imputation to impute missing values for FPL (20). The FPL is based on poverty thresholds issued by the US Census Bureau each January (21). The selection of covariates was guided by previous NSCH research on SSBs (22).

We used descriptive statistics to evaluate the proportion of children who had a caregiver-reported cavity in the past 12 months, overall, by SSB consumption, and by sociodemographic characteristics. We performed χ^2^ tests to determine the association between SSB consumption and presence of a caregiver-reported cavity, overall and by sociodemographic characteristics. We used multivariable logistic regression to estimate adjusted odds ratios (AORs) and 95% CIs for the presence of a caregiver-reported cavity by frequency of SSB consumption, controlling for sociodemographic covariates. We used SAS version 9.4 (SAS Institute Inc) and its SAS-callable version of SUDAAN software version 11 (RTI International) to conduct adjusted analysis. We used survey procedures to account for the sampling weights provided by NSCH and weighting and imputation (23) to account for nonresponse.

## Results

Among the 23,023 US children aged 1 to 5 years who had seen an oral health provider in the past 12 months, 46.0% were aged 1 to 3 years, 26.4% were aged 4 years, and 27.6% were aged 5 years (Table 1); 49.1% were female, and 48.2% were non-Hispanic White. Children of college graduates made up 57.5% of the sample, and 40.5% of children lived in a household with income at or above 350% FPL (Table 1).

**Table 1:** Proportion of Children Who Were Seen by an Oral Health Provider in the Past 12 Months and Who Had a Caregiver-Reported Cavity in the Past 12 Months (N = 23,023), by Sociodemographic Characteristics, National Survey of Children’s Health, 2021–2022

Characteristic	Caregiver-reported cavity in the past 12 months			
	All, no. (%)^a^	No, (%) SE	Yes, % (SE)	*P* value^b^
**Total**	23,023	88.4 (0.5)	11.6 (0.5)	—
**Child’s age**				
1–3 years (12–47 months)	10,423 (46.0)	93.9 (0.5)	6.2 (0.5)	<.001
4 years (48–59 months)	6,029 (26.4)	86.3 (1.1)	13.7 (1.1)	
5 years (60–71 months)	6,571 (27.6)	81.4 (1.1)	18.6 (1.1)	
**Child’s sex**				
Male	11,733 (50.9)	88.2 (0.7)	11.9 (0.7)	.57
Female	11,290 (49.1)	88.7 (0.7)	11.3 (0.7)	
**Child’s race and ethnicity**				
Hispanic	3,339 (28.3)	85.5 (1.3)	14.5 (1.3)	.03
Non-Hispanic Black	1,220 (12.1)	89.8 (1.3)	10.2 (1.3)	
Non-Hispanic White	15,274 (48.2)	89.8 (0.5)	10.3 (0.5)	
Non-Hispanic Other race^c^	3,190 (11.5)	88.7 (1.0)	11.3 (1.0)	
**Highest level of education among adults in the household**				
High school graduate/GED or less	2,739 (24.6)	82.7 (1.4)	17.3 (1.4)	<.001
Some college or technical school	4,258 (17.9)	85.2 (1.1)	14.8 (1.1)	
College degree or higher	16,026 (57.5)	91.9 (0.5)	8.1 (0.5)	
**Household income as a percentage of FPL^d^**				
<130%	3,377 (24.3)	83.8 (1.4)	16.3 (1.4)	<.001
130% to <350%	7,575 (35.3)	87.0 (0.9)	13.1 (0.9)	
≥350%	12,071 (40.5)	92.5 (0.5)	7.5 (0.5)	

Abbreviations: FPL, federal poverty level; GED, General Educational Development certificate.

a Percentages were weighted based on National Survey of Children’s Health methodology and guidance (18); percentages may not add to 100 because of rounding.

b *P* values calculated by using χ^2^ tests.

c Includes non-Hispanic Asian, American Indian/Alaska Native, Native Hawaiian and other Pacific Islander, and multiple races.

d The FPL is based on poverty thresholds issued by the US Census Bureau each January (21).

Overall, 11.6% of children had a caregiver-reported cavity in the past 12 months (Table 1). By age group, 18.6% of children aged 5 years, 13.7% of children aged 4 years, and 6.2% of children aged 1 to 3 years had a caregiver-reported cavity. More than 1 in 10 children (16.3%) who lived in a household with income less than 130% FPL had a caregiver-reported cavity, compared with 13.1% of children whose household income was 130% FPL to less than 350% FPL and 7.5% of children at or above 350% FPL.

Caregiver report indicated that 37.3% of children aged 1 to 5 years drank no SSBs in the past week, ranging from 28.0% of children aged 5 years to 46.0% of children aged 1 to 3 years (Table 2). In contrast, 62.7% of children did drink at least 1 SSB in the past week. Overall, 39.5% drank SSBs 1 to 3 times in the past week, and 23.2% drank SSBs 4 or more times in the past week. By age, the proportion who drank SSBs 4 or more times was 25.6% of 5-year-olds, 27.1% of 4-year-olds, and 19.6% of children aged 1 to 3 years (*P* < .001) (Table 2). The proportion of children who drank SSBs 4 or more times in the past week was 35.9% among children who lived in a household with income less than 130% FPL, 25.2% among those whose household income was 130% FPL to less than 350% FPL, and 13.9% for those at or above 350% FPL (*P* < .001) (Table 2).

**Table 2:** Proportion of Children Aged 1–5 Years Who Consumed Sugar-Sweetened Beverages in the Past Week, By Sociodemographic Characteristics, National Survey of Children’s Health, 2021–2022

Characteristic	Sugar-sweetened beverage intake^a^				
	All, no. (%)^b^	None in past week, % (SE)	1–3 Times in past week, % (SE)	≥4 Times in past week, % (SE)	*P* value^c^
**Total**	23,023	37.3 (0.7)	39.5 (0.7)	23.2 (0.7)	—
**Child’s age**					
1–3 years (12–47 months)	10,423 (46.0)	46.0 (1.2)	34.4 (1.1)	19.6 (1.1)	<.001
4 years (48–59 months)	6,029 (26.4)	31.9 (1.2)	41.0 (1.4)	27.1 (1.5)	
5 years (60–71 months)	6,571 (27.6)	28.0 (1.1)	46.4 (1.3)	25.6 (1.2)	
**Child’s sex**					
Male	11,733 (50.9)	37.1 (1.0)	39.6 (1.0)	23.3 (1.0)	.97
Female	11,290 (49.1)	37.5 (1.0)	39.3 (1.0)	23.2 (1.0)	
**Child’s race and ethnicity**					
Hispanic	3,339 (28.3)	28.2 (1.5)	39.6 (1.9)	32.2 (1.9)	<.001
Non-Hispanic Black	1,220 (12.1)	26.8 (2.3)	41.5 (2.5)	31.8 (2.3)	
Non-Hispanic White	15,274 (48.2)	44.3 (0.8)	38.7 (0.8)	16.9 (0.6)	
Non-Hispanic Other race^d^	3,190 (11.5)	41.2 (1.7)	40.2 (1.8)	18.6 (1.4)	
**Highest level of education among adults in the household **					
High school graduate/GED or less	2,739 (24.6)	20.1 (1.5)	41.9 (2.1)	38.0 (2.1)	<.001
Some college or technical school	4,258 (17.9)	30.2 (1.5)	40.9 (1.6)	28.9 (1.6)	
College degree or higher	16,026 (57.5)	46.8 (0.8)	38.0 (0.8)	15.2 (0.6)	
**Household income as a percentage of FPL^e^**					
<130%	3,377 (24.3)	22.2 (1.4)	41.9 (2.0)	35.9 (2.0)	<.001
130% to <350%	7,575 (35.3)	34.5 (1.3)	40.4 (1.3)	25.2 (1.2)	
≥350%	12,071 (40.5)	48.8 (1.0)	37.3 (1.0)	13.9 (0.8)	

Abbreviations: FPL, federal poverty level; GED, General Educational Development certificate.

a Caregivers were asked, “During the past week, how many times did this child drink sugary drinks?”

b Percentages were weighted based on National Survey of Children’s Health methodology and guidance (18); percentages may not add to 100 because of rounding.

c *P* values calculated by using χ^2^ tests.

d Includes non-Hispanic Asian, American Indian/Alaska Native, Native Hawaiian and other Pacific Islander, and multiple races.

e The FPL is based on poverty thresholds issued by the US Census Bureau each January (21).

**Figure 2:**
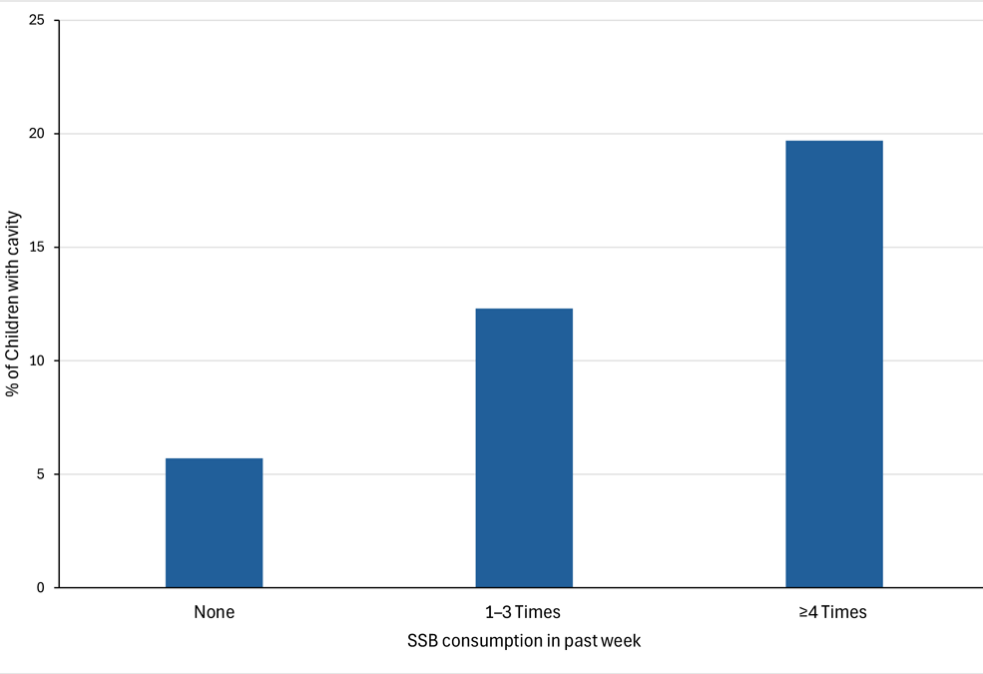
Proportion of children aged 1 to 5 years who had a caregiver-reported cavity in the past 12 months, by consumption of SSBs in the past week (N = 23,023), National Survey of Children’s Health, 2021–2022. Abbreviation: SSB, sugar-sweetened beverage.

Approximately one-fifth (19.7%) of children aged 1 to 5 years who were reported to drink SSBs 4 or more times in the past week had a caregiver-reported cavity, compared with 12.3% of children who drank SSBs 1 to 3 times in the past week and 5.7% of children who drank none in the past week (Figure 2). Children who drank SSBs 4 or more times in the past week had 4.0 (95% CI, 3.1–5.2) times higher unadjusted odds and 2.8 (95% CI, 2.1–3.6) times higher adjusted odds of a caregiver-reported cavity than those who drank no SSBs (Table 3). Children who drank SSBs 1 to 3 times in the past week had 2.3 (95% CI, 1.8–2.9) times higher unadjusted odds and 1.7 (95% CI, 1.4–2.2) times higher adjusted odds of a caregiver-reported cavity than those who drank no SSBs (Table 3).

**Table:** 

SSB consumption in past week	Children with cavity, %
None	5.7
1 to 3 Times	12.3
≥4 Times	19.7

**Table 3:** Association Between Caregiver-Reported Cavities in the Past 12 Months and SSB Consumption Among Children Aged 1–5 Years, National Survey of Children’s Health, 2021–2022

Consumption of SSBs	Presence of a caregiver-reported cavity in the past 12 months	
	Unadjusted OR (95% CI)	Adjusted OR^a^ (95% CI)
None	1 [Reference]	1 [Reference]
1–3 times in past week	2.3 (1.8–2.9)	1.7 (1.4–2.2)
≥4 times in past week	4.0 (3.1–5.2)	2.8 (2.1–3.6)

Abbreviation: SSB, sugar-sweetened beverage; OR, odds ratio.

a Adjusted for age, sex, race and ethnicity, highest level of education among adults in the household, and household income as a percentage of the federal poverty level.

## Discussion

Nationally representative survey data for very young US children (aged 1–5 years) in 2021–2022 showed that frequent SSB consumption was common: 62.7% were reported by their caretaker to have consumed at least 1 SSB in the past week. SSB consumption was significantly associated with caregiver-reported cavities in early childhood. Both the prevalence and adjusted odds of having cavities increased with frequency of SSB consumption.

Children aged 1 to 3 years consumed SSBs infrequently relative to children aged 4 and 5 years and had the lowest prevalence (6.2%) of caregiver-reported cavities. However, any SSB consumption at 1 year of age is inconsistent with the 2020–2025 *Dietary Guidelines for Americans* recommendation to avoid added sugars; these guidelines also recommend limiting consumption of added sugars among children aged 2 or 3 years (11). Children aged 4 or 5 years in our study were more likely than children aged 1 to 3 years to consume SSBs at least once in the past week and have a caregiver-reported cavity in the past year. Caregiver-reported cavities and prevalence of consuming SSBs differed significantly by age. Nearly one-fifth (18.6%) of children aged 5 years had a caregiver-reported cavity, and nearly three-quarters (72.0%) consumed at least 1 SSB in the past week. Furthermore, children who drank SSBs 4 or more times in the past week had 2.8 higher adjusted odds of having a cavity than children who did not drink SSBs. These results echo conclusions from a similar study that used NHANES 2011–2014 data (10) to show increased odds of cavities with SSB consumption in very young children.

Early childhood is an important period to prevent cavities and for caregivers to help initiate preventive oral health care (24). In addition to limiting added sugars, it is also important to maintain good oral hygiene practices and receive routine oral health care, including preventive services, such as fluoride varnish starting by 1 year of age, to prevent cavities (25,26).

Our data showed that more than 60% of children aged 1 to 5 years drank an SSB at least once in the past week. Public health interventions can help to decrease SSB consumption (27–30). A public health campaign in a Maryland county achieved a 30% decrease in soda (soft drink) sales and an increase in water sales across the population by engaging the community, using media, and focusing on policy change (27). Data collected in the United Kingdom in 2017 showed that warning labels may be an option to reduce parents’ selection of SSBs for their children aged 11 to 16 years (28). In that study, labels with images of disease processes caused by SSBs, such as cavities, had lower odds of being selected by parents (28). Studies conducted after implementation of SSB taxes have also demonstrated potential oral health benefits (29,30). A study in the United Kingdom demonstrated a 28.6% reduction in hospitalizations for carious tooth extractions among children aged 1 to 4 years and a 5.5% reduction among children aged 5 to 9 years after implementation of an SSB tax in 2016 (29). Public health efforts coupled with individual oral health behaviors starting in childhood might help to reduce SSB consumption and prevent cavities in very young children.

To further reduce SSB consumption among children, several environmental interventions could be implemented. A systematic review published in 2020 showed that such interventions can lower SSB consumption (31). Enhancing in-store promotion of healthier beverages in supermarkets, using home-based interventions to improve the availability of healthier beverages, and equipping food benefit programs with incentives for purchasing healthy food are behavioral design strategies that could have a moderate effect on family choices (32). Efforts to address SSB consumption could affect health outcomes beyond oral health. For example, frequent SSB consumption is a risk factor for childhood obesity, and decreased consumption of sugar at a very young age could reduce the risk of chronic diseases in adulthood (32,33).

### Limitations

Our study has several limitations. First, this study excluded children who had not seen an oral health provider in the past 12 months; some of these children might have had cavities but did not have the opportunity for diagnosis due to lack of oral health care. Of note, nearly 80% of the children excluded due to lack of dental care were aged 1 to 3 years, and 50% did not drink SSBs in the past week. Second, NSCH is a cross-sectional survey; thus, causal associations between SSB consumption and cavities could not be discerned. Third, NSCH data are reported by caregivers, subjecting these data to biases, including recall, information, and social desirability biases. These biases might have led to underestimation of the frequency of SSB consumption; for example, caregivers may have reported fewer SSBs than their child actually drank (34). Caregiver characteristics, such as education and socioeconomic status, could also influence reporting accuracy, leading to overestimating or underestimating of SSBs consumed (34). Fourth, this study was unable to account for other preventive oral health practices, such as how consistently and thoroughly children brushed and flossed. Fifth, the prevalence of cavities could have been overestimated if caregivers reported instances of early demineralization without cavitation. Finally, the study could not account for other sugary foods consumed by children, such as baked goods, cereals, or candy, that could also affect their risk of cavities. Although this study had limitations, it had multiple strengths, including national representativeness and the assessment of SSB consumption and cavities in a very young age group.

### Conclusion

Future research could explore the long-term effects of early SSB consumption on oral health outcomes beyond cavities, such as periodontal disease. Additionally, examining data on health insurance status, type of health insurance, or dietary factors, such as carbohydrate consumption, could provide details for tailored public health interventions. Finally, further examining the oral health and dietary behaviors of children with and without dental visits might help us better understand the implications of access to care on oral health outcomes.

Recommendations exist to limit or avoid sugar intake by very young children and seek regular dental care for them. However, based on our current data, frequent SSB consumption among very young children is common, with more than 60% of children aged 1 to 5 years consuming an SSB at least once in the past week. Caregiver-reported cavities were associated with frequent SSB consumption in early childhood: the more SSBs consumed, the more likely children were to have a cavity. These data can further amplify existing recommendations for promoting healthy dietary and preventive behaviors during early childhood. They can be used by public health and oral health providers to enhance messaging for caregivers about reducing or eliminating SSBs in their children’s diets and promoting good oral hygiene for children, including regular preventive dental visits to prevent poor oral health outcomes. Ultimately, finding creative strategies to reduce SSB consumption among very young children and encouraging children and their caregivers to make healthier choices can mitigate oral disease.
